# Supplementary material for: A Global Screen for Assembly State Changes of the Mitotic Proteome by SEC-SWATH-MS
Source: Cell Syst. 2020 Feb 26;10(2):133–155.e6. doi: 10.1016/j.cels.2020.01.001 (PMC7042714; doi:10.1016/j.cels.2020.01.001)

Q9UIW2 | PLXA1_HUMAN | PLXNA1 NOV PLXN1
Monomer MW [kDa]: 211.067 Monomer expected elution fraction: 34

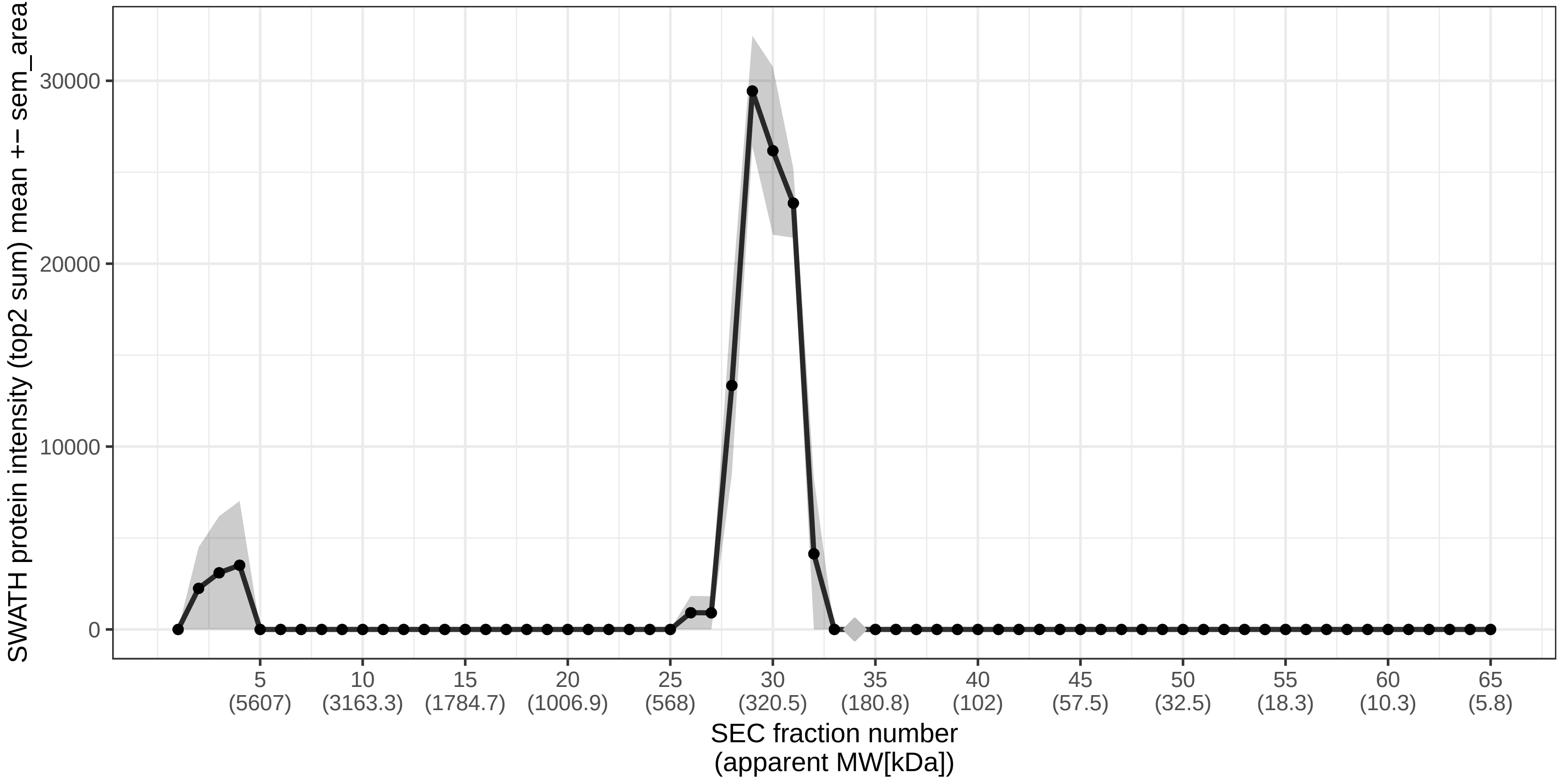

Supplement: Data S1. SEC-SWATH-MS Protein Chromatograms, Related to Figure 1 [file mmc6.zip › SECchrom_Q9UIW2_PLXA1_HUMAN_PLXNA1_NOV_PLXN1.pdf]
